# What is vitamin A and why do we need it?

**Published:** 2013

**Authors:** Clare Gilbert

**Affiliations:** Co-director: International Centre for Eye Health, Disability Group, London School of Hygiene and Tropical Medicine, London, UK.

**Figure:**
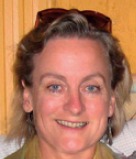
Clare Gilbert

Vitamin A, along with other vitamins, minerals and other compounds, is an essential micronutrient. This means that our bodies cannot manufacture it and therefore it has to be included in our diet.

Vitamin A from food is stored in the liver until required by the body and is bound to protein before being transported to where it is needed.

Vitamin A is essential for many physiological processes, including maintaining the integrity and function of all surface tissues (epithelia): for example, the skin, the lining of the respiratory tract, the gut, the bladder, the inner ear and the eye. Vitamin A supports the daily replacement of skin cells and ensures that tissues such as the conjunctiva are able to produce mucous and provide a barrier to infection. Vitamin A is also essential for vision under conditions of poor lighting, for maintaining a healthy immune system, for growth and development and for reproduction. Vitamin A supports many systems in the body. For this reason, vitamin A deficiency is now referred to as vitamin A deficiency disorders. For simplicity, however, we will continue to use the older term vitamin A deficiency (VAD).

One of the main consequences of VAD is an increased risk of severe infection. Infection increases the body's demand for vitamin A and so the deficiency gets worse. Children can therefore become involved in a vicious cycle of deficiency and infection, which is why vitamin A deficiency is such an important cause of child mortality.

**Food sources of vitamin A**

**Fruits and vegetables**

Dark green leafy vegetables, for example amaranth (red or green), spinach and chard

Orange-fleshed sweet potatoes

Carrots

Squashes/pumpkins

Yellow maize

Mangoes

Papayas

**Animal sources**

Liver, eggs, milk (including breast milk)

**Oils**

Red palm oil or biruti palm oil

**Figure:**
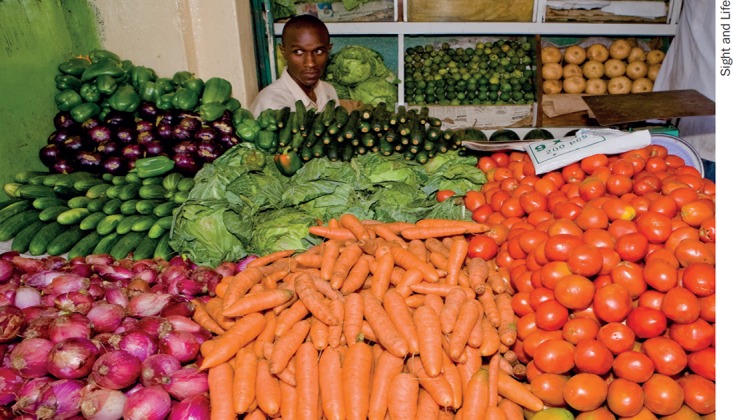
Vitamin A, whether from plant or animal sources, is essential for health

## Sources of vitamin A

There are two main sources of vitamin A: animal sources and plant sources. All the sources of vitamin A need some fat in the diet to aid absorption.

In animal sources, vitamin A is found as retinol, the ‘active’ form of vitamin A. Liver, including fish liver, is a very good source. Other animal sources are egg yolk (not the white) and dairy products such as milk (including human breast milk), cheese and butter. Meat, from the animal's muscles, is not a good source.

Plant sources contain vitamin A in the form of carotenoids which have to be converted during digestion into retinol before the body can use it. Carotenoids are the pigments that give plants their green colour and some fruits and vegetables their red or orange colour.

Plant sources of vitamin A include: mangos, papaya, many of the squashes, carrots, sweet potatoes and maize (but not the white varieties). Other good sources of vitamin A are red palm oil and biruti palm oil. (Note: if these oils are boiled to remove their colour the vitamin A is destroyed.)

Some fruits and vegetables are easier to digest than others, and it has been shown that dark green leafy vegetables such as spinach or amaranth are harder to digest. Mashing these vegetables up after cooking makes them easier to digest. When mashed they can be added to staples, which also makes them easier to disguise – children the world over do not like green vegetables!

It is important that all sources of vitamin A are not overcooked, as this can reduce the vitamin A content. Ultraviolet light can also reduce the vitamin A content of food, so drying of fruits such as mangos should not be done in direct sunlight (see page 73).

Diets that rely heavily on local carbohydrates, such as rice, fufu, ugali, cassava, millet and sorghum, are very low in vitamin A, unless vitamin A-rich foods are added.

## How much vitamin A does a child need?

Because children are growing, they need a relatively high intake of vitamin A; about half as much as an adult. Another reason for the relatively high intake is because children are prone to infection which increases the metabolic rate and hence the rate at which they use vitamin A.

Breast milk contains enough vitamin A for children up to six months of age, but after that complementary foods (the foods given in addition to breast milk) should include small amounts of vitamin A-rich foods.

For a young child, a balanced diet that is rich in vitamin A should include helpings of at least 2–3 vitamin A-rich fruits and vegetables a day, plus a little bit of fat to aid absorption.

Young children are totally dependent on their mother or other carers for their diet, and so it is vital that mothers and carers of young children know what constitutes a healthy diet for their child.

*With thanks to Temina Mkumbwa, Christina Nyhus Dhillon, Heather Katcher, and Jessica Blankenship*.

